# MOF-Derived CeO_2_ and CeZrO_x_ Solid Solutions: Exploring Ce Reduction through FTIR and NEXAFS Spectroscopy

**DOI:** 10.3390/nano13020272

**Published:** 2023-01-09

**Authors:** Davide Salusso, Silvia Mauri, Gabriele Deplano, Piero Torelli, Silvia Bordiga, Sergio Rojas-Buzo

**Affiliations:** 1 Department of Chemistry, NIS Center and INSTM Reference Center, University of Turin, 10125 Turin, Italy; 2 European Synchrotron Radiation Facility, CS 40220, CEDEX 9, 38043 Grenoble, France; 3 IOM CNR Laboratorio TASC, AREA Science Park, Basovizza, 34149 Trieste, Italy; 4 Department of Physics, University of Trieste, Via Valerio 2, 34127 Trieste, Italy

**Keywords:** MOFs-derived oxides, Ce-UiO-66, CeZr-UiO-66, CeO_2_, CeZrO_x_, Ce^3+^ quantification, FT-IR, AP-NEXAFS

## Abstract

The development of Ce-based materials is directly dependent on the catalyst surface defects, which is caused by the calcination steps required to increase structural stability. At the same time, the evaluation of cerium’s redox properties under reaction conditions is of increasing relevant importance. The synthesis of Ce-UiO-66 and CeZr-UiO-66 and their subsequent calcination are presented here as a simple and inexpensive approach for achieving homogeneous and stable CeO_2_ and CeZrO_x_ nanocrystals. The resulting materials constitute an ideal case study to thoroughly understand cerium redox properties. The Ce^3+^/Ce^4+^ redox properties are investigated by H_2_-TPR experiments exploited by in situ FT-IR and Ce M_5_-edge AP-NEXAFS spectroscopy. In the latter case, Ce^3+^ formation is quantified using the MCR-ALS protocol. FT-IR is then presented as a high potential/easily accessible technique for extracting valuable information about the cerium oxidation state under operating conditions. The dependence of the OH stretching vibration frequency on temperature and Ce reduction is described, providing a novel tool for qualitative monitoring of surface oxygen vacancy formation. Based on the reported results, the molecular absorption coefficient of the Ce^3+^ characteristic IR transition is tentatively evaluated, thus providing a basis for future Ce^3+^ quantification through FT-IR spectroscopy. Finally, the FT-IR limitations for Ce^3+^ quantification are discussed.

## 1. Introduction

Ce-based catalysts are of great interest for their redox properties in a wide range of catalytic reactions such as CO oxidation, CO_2_ hydrogenation, water–gas shift, and many more [1,2,3,4,5,6,7,8,9,10]. The catalyst surface reactivity is strongly dependent on the presence of coordinatively unsaturated sites (CUS) [11,12,13]. The CUS can be simply described as surface defects where the site instability provides a higher energy state, enhancing the target reaction. The CUS concentration is synthesis-dependent since high-temperature calcination treatments induce crystallite sintering and a consequent loss of defects [14]. Nevertheless, most of the investigated reactions occur at elevated temperatures or pressures, which require a certain degree of catalyst stability. The synthesis of the catalyst should therefore require a simple preparation procedure which at the same time provides a high number of defective sites which are also stable at the operating temperatures of the catalyst.

The commonly used sol–gel methods generally involve long and complex synthesis procedures [4,15,16,17]. On the contrary, direct precursor calcination has shown a great potential for the direct preparation of catalysts [18,19,20,21,22]. In the latter approach, promising results have been reported for direct calcination of metal–organic frameworks (MOFs) at moderate temperatures (300–500 °C) [20,21,22,23,24,25,26,27,28,29]. Indeed, depending on the employed linkers, MOF synthesis can be very simple and cheap [30]. Furthermore, the natural separation of the oxide-based clusters by organic ligands prevents crystals from sintering even at high calcination temperatures, thus preserving the surface defects of the catalysts. It is noteworthy that Ce-based MOFs were recently studied as replacements for oxides in several chemical reactions [31,32,33]. However, the MOFs’ stability still limits their practical applications [34,35]. For this reason, MOF calcination is still preferred for preparation of stable nanocatalysts. Considering Ce-based oxides, while CUS reduce the activation energy of the reaction, the key catalytic redox role is usually played by Ce^3+/4+^ interconversions [1,2,12,13]. For this reason, tracking the cerium oxidation state is of major interest for understanding catalytic mechanisms. The oxidation state of cerium is often monitored by electron paramagnetic resonance (EPR) or X-ray-based techniques such as photoelectron spectroscopy (XPS), absorption spectroscopy (XAS) and near-edge absorption fine structure (NEXAFS) [36,37,38,39,40]. However, from a catalytic viewpoint, only XAS spectra collected with hard X-rays at Ce K- or L_3_-edges can access Ce^3+^/Ce^4+^ ratios under high temperature and pressure conditions. Unfortunately, these measurements are limited to synchrotron sources, which limits their availability. However, the presence of Ce^3+^ can also be identified with the less expensive/more available infrared spectroscopy. Indeed, it is well known that the Ce^3+^ 4f ground state splits into doublet ^2^F_5/2_ and ^2^F_7/2_ energy levels. They are separated by about 2000 cm^−1^ and the ^2^F_5/2_→^2^F_7/2_ electronic transition is observed in the infrared range at 2127 cm^−1^ [14,41,42,43,44,45,46]. The presence/absence of this absorption band was then related to the occurrence of Ce^3+^ and it has been recently used to qualitatively monitor cerium reduction in a NiCeO_2_ sample [18]. From the infrared viewpoint, the hydroxyl stretching vibration could also be used to selectively monitor Ce^3+^ formation on the catalyst surface. In fact, the ν(OH) position (≈3600 cm^−1^) depends on the hydroxyl-cation bond order, which is directly affected by Ce oxidation state, i.e., Ce^3+^ increases the bond order, causing a hypsochromic shift of the vibration [47]. The ν(OH) frequency can then be used to identify the formation of Ce^3+^-V_O_ sites on the catalyst first surface layer, which is inaccessible to any other X-ray techniques as it represents a penetration depth of at least few nm. The use of infrared spectroscopy to safely monitor cerium’s oxidation state would then provide an incredible boost to redox mechanism evaluation since FT-IR and DRIFT cells capable of operating under several thermochemical conditions are now available [48,49,50].

In this work, we have then prepared three MOF samples with the UiO-66 structure and different Ce:Zr ratios on the clusters, i.e., 100% Ce, 50:50 Ce:Zr and 5:95 Ce:Zr. The three samples were calcined under aerobic conditions at 450 °C to obtain three stable and defective oxides containing the respective Ce:Zr ratio. FT-IR spectra of the three CeO_2_ and CeZrO_x_ derived-oxides were recorded during temperature programmed oxidation/reduction experiments to monitor ν(OH) and Ce^3+^ infrared bands. Moreover, to compare and quantify Ce^3+^ evolution, the same experiment was repeated with an ambient pressure NEXAFS set-up. Ce M_5_-edge NEXAFS spectra were recorded under in situ conditions and Ce^3+^ was quantified through MCR-ALS routine. Ce^3+^ quantification was then combined with Ce^3+^ IR absorbance to determine its infrared molar absorption coefficient.

## 2. Materials and Methods

### 2.1. Samples Preparation

The MOF syntheses were carried out following a procedure described in the literature [51]. The corresponding amounts of aqueous solutions of cerium(IV) ammonium nitrate (Sigma-Aldrich, ≥99.99%) and/or zirconium(IV) dinitrate oxide hydrate (Sigma-Aldrich, 99%) (0.53 M) were added to a Pyrex reactor containing terephthalic acid (Sigma-Aldrich, 98%) (260 mg) and N,N-dimethylformamide (DMF) (VWR Chemicals, ≥99.8%) (see Table S1). Finally, and only in the case of Ce/Zr-UiO-66 materials, a known amount of formic acid (Sigma-Aldrich, 98%) (2.07 mL) was employed as a modulator. The resulting mixtures were magnetically stirred at 100 °C for 15 min. Then, the glass vessel reactors were cooled to RT and the reaction medium was collected by centrifugation. Finally, the MOFs were washed three times with DMF and twice with acetone. The as-obtained materials were allowed to dry at RT overnight prior to the analyses. 

The MOF-derived materials were obtained by a thermal treatment under aerobic conditions. The corresponding amount of the MOF (Table S2) was calcined up to 450 °C with a ramp of 5 °C/min with a total flow of 0.5 mL/min (air). This temperature was maintained for 4 h to completely remove the organic components. 

### 2.2. Thermogravimetric (TG) Analysis

The TG profile was collected with a TA Instruments Q600 thermobalance under an air flow (100 mL/min) with a ramp of 5 °C/min from RT to 600 °C with about 5 mg of sample in an alumina crucible. 

### 2.3. Powder X-ray Diffraction (PXRD) 

PXRD patterns were collected with a Panalytical X-Pert diffractometer in the 3–50° and 10–100° 2θ range for UiO-66 and oxides samples, respectively. The crystallite size was extracted through peak shape refinement using Thompson–Cox–Hastings (TCH) function implemented in FullProf software [52,53]. 

### 2.4. Specific Surface Area (SSA) 

SSA was determined by applying the Brunauer–Emmett–Teller (BET) equation to N_2_ adsorption/desorption isotherms collected at 77 K obtained with a Micromeritics ASAP 2020 physisorption analyzer. The samples were previously evacuated at 120 °C (for the UiO-66 samples) and 400 °C (for oxides). 

### 2.5. Transmission Electron Microscopy (TEM)

TEM was exploited to obtain morphological and structural information of the samples. The analyses were carried out by using a TEM Jeol JEM 3010 UHR (300 kV, LaB_6_ filament) equipped with X-ray EDS analysis by a Link ISIS 200 detector. The samples, in the form of powders, were deposited on a Cu grid coated with a porous carbon film. 

### 2.6. In Situ Fourier Transform-Infrared (FT-IR) 

FT-IR spectra were collected with an Aabspec cell suitable for thermal treatments under gas flows. The cell was mounted in a Bruker Invenio R spectrophotometer. Spectra were collected in transmission mode in the 4000–500 cm^−1^ range with 2 cm^−1^ resolution. CeO_2_ was pressed in a self-supported pellet of area ≈ 10 cm^2^. The pellet was held in a gold envelope and placed in the cell sample holder. The measurement protocol (Figure S1) consisted of two parts: (I) The CeO_2_ surface was cleaned from adsorbed species (H_2_O, carbonates, etc.) by heating the pellet from RT to 400 °C (5 °C/min) under 50 mL/min of N_2_ (99.9999%):O_2_ (99.99999%) (1:1) stream. The temperature was then held at 400 °C for 60 minutes and then cooled to RT. To prevent self-reduction, the oxidising gas mixture was maintained until 150 °C, while from 150 °C to RT, the gas stream consisted of pure N_2_ only. (II) Depending on the performed temperature programmed oxidation (TPO, Figure S1a) or reduction (TPR, Figure S1b) experiment (i.e., O_2_-TPO or H_2_-TPR, respectively), the gas mixture was replaced with a N_2_:O_2_ (99.9999%) or N_2_:H_2_ stream at 25 °C and held for 15′. After that, the TPO or TPR experiment was performed by heating the pellet from RT to 300 °C at 5 °C/min rate with a final holding at 300 °C for 30′. Both measurements were performed on the same pellet to guarantee experimental reproducibility.

### 2.7. Ambient Pressure Near-Edge X-ray Absorption Spectra (AP-NEXAFS) 

AP-NEXAFS spectra were measured at APE-HE beamline of the Elettra Italian Synchrotron radiation source. CeO_2_ was placed in a specially designed reactor cell allowing thermal treatments in the RT–400 °C range under a gas atmosphere of 1 bar. The total electron yield (TEY) mode was used to record the experimental spectra. Ce M_5_-edge spectra were collected from 880 to 910 eV with 0.01 eV energy resolution. The measurement protocol followed the same steps as described for the in situ FTIR measurements (Figure S1) with N_2_ replaced by He (99.99999%) and with the maximum temperature limited to 350 °C. Spectra were energy aligned to a reference CeO_2_ measured simultaneously with the MOF-derived material. Spectra were background subtracted and energy aligned with the Thorondor software [54]. A 6th order polynomial was used for background subtraction. Ce^3+^/Ce^4+^ spectral pure components and their concentration evolution were extracted using MCR-ALS implemented in MATLAB. The MCR-ALS protocol lead to lack of fit (LOF) of 3.9% with PCA and 6.2% with experimental spectra, with 99.6% of variance explained [55]. Spectra and concentration were constrained to positive values while the closure condition was applied to concentrations. Notably, to increase the variance between spectra, the H_2_-TPR was conducted until 350 °C to improve Ce^4+^/Ce^3+^ spectra separation. Moreover, to guarantee reproducibility of the MCR-ALS protocol for the three samples, the collected spectra were analysed together in the same dataset. Ten replicas of CeO_2_ and CeF_3_ reference spectra were added at the end of the dataset to support the MCR-ALS protocol in finding Ce^4+^ and Ce^3+^ pure spectra components.

## 3. Results and Discussion

**Figure 1:**
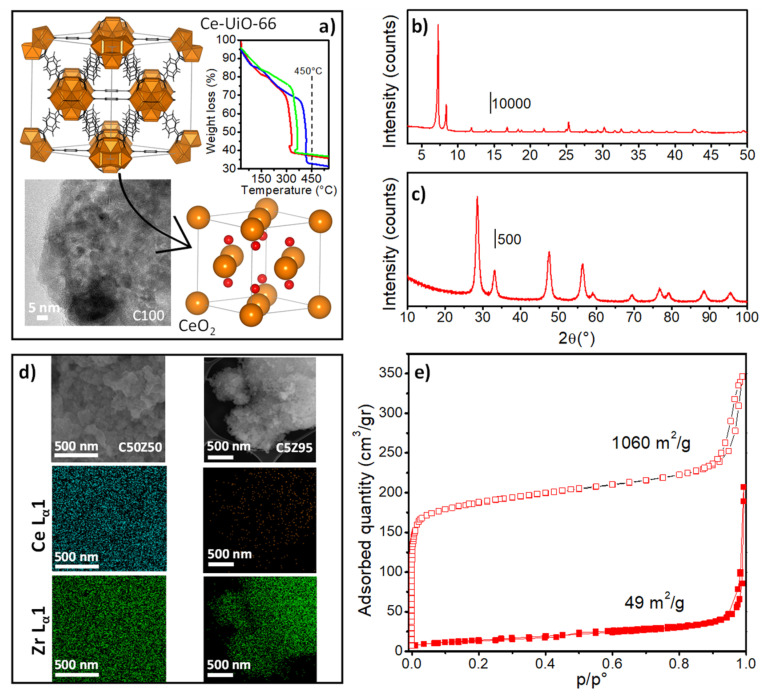
(**a**) Reported Ce-UiO-66 and CeO_2_ structures (Ce atoms/clusters in orange, O in red). TG analysis of C100-(red line), C50Z50-(green line), and C5Z95-UiO-66 (blue line) are shown in the top inset. The C100 TEM image is shown in the bottom inset. The PXRD pattern of (**b**) C100-UiO-66 and (**c**) C100 samples. (**d**) C50Z50 and C5Z95 TEM images and EDX maps. (**e**) N_2_ adsorption–desorption isotherms collected at 77 K over C100-UiO-66 (empty squares) and C100 (full squares) samples.

Ce-UiO-66 and CeZr-UiO-66 were synthesized following the procedure described by Lammert et al. [56] and reported in Materials and Methods section. The resulting solids showed the *fcu* topology characteristic of the UiO-66 materials (Figure 1a), as it can be deduced from the PXRD patterns reported in Figure 1b and Figure S2a. The PXRD patterns also presented a shift in Bragg reflections towards higher 2θ values with Zr concentration (Figure S2b), in line with the smaller ionic radii of Zr^4+^ (0.84 vs. 0.97 Å of Ce^4+^) [57]. On the other hand, the N_2_ adsorption isotherms revealed the microporous nature of these MOFs. The evaluated SSA was 1000–1440 m^2^/g, in line with the values reported in the literature (Figure S4 and Table S3) [56]. Thermogravimetric (TG) analysis (Figure 1a) showed ~40% of weight loss in the 300–500 °C temperature range, which corresponded to the degradation of the organic linker and the subsequent transformation of the MOFs into the metal oxide. The increase in the onset temperature with the Zr content was related to the known higher stability of pure Zr-UiO-66 [58]. MOF calcination was then conducted at 450 °C to eliminate the organic components, in line with the temperatures range reported in the literature [26,27,28,29]. C100-UiO-66 calcination could have been conducted at lower temperature (≈350 °C); however, this would have induced an inhomogeneity in the samples’ thermal treatments. In fact, all the successive measurements applied heating steps up to 400 °C. The calcination of the three MOFs at 450 °C then guaranteed the derived-oxides’ stability within the RT–400 °C temperature range. The PXRD pattern of the obtained yellowish powder (Figure 1c) presented Bragg peaks ascribable to a cubic (Fm-3m) CeO_2_ phase (JCPDS file number 34–394). As for the initial MOFs, Bragg reflections shifted to higher 2θ values with Zr concentration, in line with the Zr^4+^/Ce^4+^ ionic radii differences (Figure S2c,d). TEM images (Figure 1a,d) and crystallite size determined by PXRD Rietveld refinement (Figure S3, Table S3) confirmed that particles of among 5–10 nm were well defined and not agglomerated. An EDX analysis (Figure 1d) unveiled that the obtained oxides maintained the MOF composition, i.e., C100 (pure CeO_2_), C50Z50 (Ce:Zr 49:51 wt%), and C5Z95 (Ce:Zr 5:95 wt%) with an homogeneous distribution of Ce and Zr on the surface of the samples, confirming solid solution formation. The obtained oxides presented a significant drop in SSA (Table S3), in line with the collapse of the UiO-66 structure. Moreover, hysteresis necks (Figure 1e and Figure S4) were not observed in any of the samples. This indicated the absence of interparticle porosity, which is in line with the non-agglomerated particles observed by microscopy results.

To obtain the best achievable information on the Ce oxidation state through FT-IR spectroscopy, O_2_-TPO and H_2_-TPR were collected over previously activated C100, C50Z50, and C5Z95 samples. The sample activation was conducted following the protocol described in the experimental section with the aim of cleaning its surface from adsorbate species (i.e., H_2_O, carbonates, and organic compounds). While the O_2_-TPO experiment was conducted to have a reference spectrum of oxidised CeO_2_ at the different temperatures, the H_2_-TPR experiment was expected to introduce Ce^3+^ and oxygen vacancies (V_O_) into the sample. Indeed, as reported in Equation (1), the exposure of *Ce*^4+^-*O*-*Ce*^4+^ sites to H_2_ at high temperatures can cause a redox reaction leading to cerium reduction and water formation.

(1) $$Ce^{4+}-O-Ce^{4+}+H_{2}\to \;Ce^{3+}-V_{O}-Ce^{3+}+H_{2}O\;\;$$

**Figure 2:**
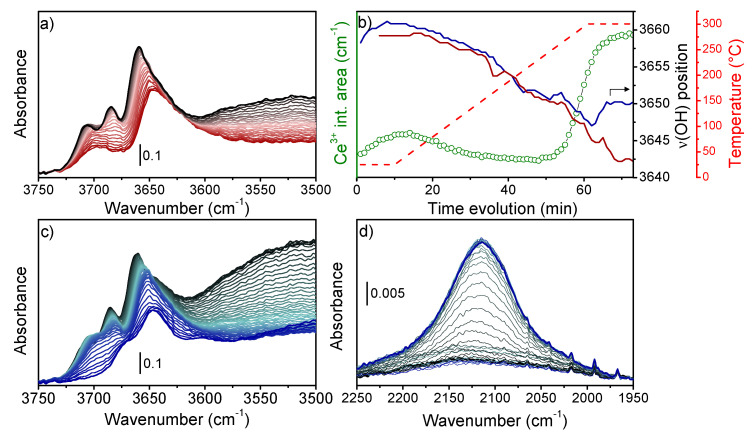
(**a**) Detail of FT-IR spectra ν(OH) region collected during the O_2_-TPO experiment (temperature rise is shown as from black to red). (**b**) Position of t-OH maximum during H_2_-TPR (blue line) and O_2_-TPO (red line) experiments compared with Ce^3+^ band integrated area (green circles) observed during the H_2_-TPR experiment. The temperature profile is reported with a dashed red line. Detail of FT-IR spectra (**c**) ν(OH) and (**d**) Ce^3+ 2^F_5/2_→^2^F_7/2_ regions (baseline corrected) collected during H_2_-TPR experiments (temperature rise is shown as from black to blue).

Even though CeO_2_ and CeZrO_x_ infrared spectra have been known for decades, we here aim to show how to exploit spectral fingerprints related to the Ce oxidation state. C100 spectra collected after thermal activation (described in SI) presented three bands in the ν(OH) region (Figure S5′) at 3704, 3684, and 3657 cm^−1^ ascribed to monodentate (m-OH), bidentate (b-OH), and tridentate (t-OH) hydroxyl groups (Figure S6), respectively. After the thermal activation, O_2_-TPO was conducted (Figure 2a) to track the reference variation in ν(OH) positions with temperature. During heating under O_2_, the absorbance of m- and b-OH bands decreased until a single broad band centered at 3696 cm^−1^ was formed. At the same time, the broad band centered at ~3500 cm^−1^, related to physisorbed water, decreased in intensity. In constrast, the t-OH lost intensity and its position shifted linearly to lower wavenumbers, (Figure 2b, red line) until it was stabilized when T = 300 °C. The band position bathochromic linear shift is associated with crystal lattice expansion. Instead, the loss of band integrated area could be related to either a decrease in surface OH groups (i.e., sample dehydration) or to a temperature dependence of the OH molar absorption coefficient (ε) [59,60]. Indeed, following the Beer–Lambert Law (Equation (2)), a variation in ε would directly affect the integrated band area. However, this can be excluded since surface dehydration was observed from the corresponding decrease in the broad band at 3500 cm^−1^. After having determined the spectral behavior under heating conditions, H_2_-TPR was conducted on the activated catalyst. First of all, by observing the physisorbed water band (~3500 cm^−1^), we noticed that the band intensity was relatively higher than the first spectra of the O_2_-TPO experiment. During H_2_-TPR, the band intensity initially decreased, indicating water desorption, while it increased again at higher temperatures. Water formation under H_2_/300 °C is the first evidence of cerium reduction with parallel formation of oxygen vacancies (V_O_), as described in Equation (1). 

Moreover, the higher intensity of the band in the spectra under H_2_/RT conditions suggested that surface reduction had already started at RT.

Considering the Ce-OH groups, m-OH and b-OH were rapidly consumed, whilst the t-OH band underwent a non-linear bathochromic shift (Figure 2c). Since t-OH presented a higher stability during thermal treatment, we focused on its band maximum position (Figure 2b, blue line). In particular, we observed that: (I) the frequency increased from 3658 cm^−1^ to 3661 cm^−1^ at T = 25 °C when the gas environment changed from N_2_ to N_2_:H_2_ (see protocol Figure S1). (II) The frequency decreased when the temperature increased to 300 °C, in line with lattice expansion, and (III) the frequency shifted to 3650 cm^−1^ (8 cm^−1^ higher than the final position reached under O_2_, i.e., 3542 cm^−1^) as soon as the temperature was stabilized at 300 °C. The origin of the t-OH shift under H_2_ can be further understood from the Ce^3+ 2^F_5/2_→^2^F_7/2_ electronic transition occurring at 2127 cm^−1^. Indeed, while this band was not observed under O_2_ (Figure S5b″), it presented a relevant intensity under H_2_ (Figure 2d). To understand the t-OH hypsochromic shift, it should be considered that during cerium reduction, *Ce*^3+^*-V_O_-Ce*^3+^ sites are formed (Equation (1)). The t-OH can then arrange over the *Ce*^3+^*-V_O_-Ce*^3+^ site, formally becoming a t’-OH group (Figure S6). Ce^3+^ increases the hydroxyl bond order causing a hypsochromic shift of the t’-OH vibration [47]. Furthermore, the Ce^3+^ integrated band absorbance intensity reported in Figure 2b (green circles) followed the same trend as the t-OH hypsochromic shift. In fact, the Ce^3+^ area (I) increased when H_2_ was added to the gas environment at a constant temperature of 25 °C, (II) it decreased during heating, and (III) it rose dramatically at T > 250 °C. This confirms the relationship between Ce^3+^ content and t-OH shift. A direct comparison of t-OH position with Ce^3+^ area gave a complete (non-quantitative) view of Ce^3+^-V_O_ formation on both the catalyst surface (ν(OH)) and in the bulk (Ce^3+^ band). Notably, the H_2_O, ν(t-OH) or Ce^3+^ band area which highlights surface cerium reduction under H_2_, showed that the reaction had already begun at RT. This is in line with cerium’s higher reducibility in the case of MOF-derived CeO_2_ samples [26,27,28,29]. 

Nevertheless, the amount of available information extractable from FTIR spectra decreased in the case of CeZrO_x_ solid solutions, i.e., C50Z50 and C5Z95. In the former, the even distribution of Ce/Zr within the lattice increased the hydroxyl species population with potential similar vibrational frequencies (see Figure S6). This caused a broadening of the observed band which prevented a precise evaluation of the t-OH shift reported in Figure S7a. On the contrary, in the C5Z95 sample, the lower Ce content reduced the broadening of the OH band. This allowed observation of the same behavior noticed for C100, i.e., m-OH was consumed, b-OH was preserved, and t-OH presented a non-linear bathochromic shift. Moreover, the maximum position of the latter presented an hypsochromic shift at T ≈ 150 °C, prevailing over the lattice expansion-induced bathochromic shift (Figure S7f). As showed by NEXAFS measurements (see discussion hereafter), C5Z95-ox already contained Ce^3+^. This indicated that at 150 °C, the Ce^3+^-V_O_ surface concentration was sufficiently high to induce the observed shift. Concerning the Ce^3+ 2^F_5/2_→^2^F_7/2_ band, the higher Ce content in the C50Z50 sample allowed observation of the band formation (Figure S7b) which highlighted that Ce^4+^ reduction began at around 250 °C (Figure S7c). On the contrary, in C5Z95, the low Ce content did not allow observation of the band (Figure S7e).

**Figure 3:**
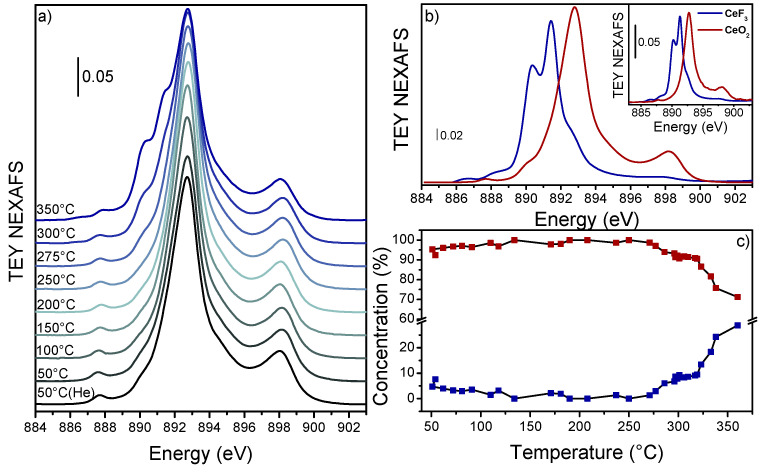
(**a**) C100 Ce M_5_-edge experimental NEXAFS spectra collected under 50 mL/min H_2_:He (3:2) from RT (black line) to 350 °C (blue line). The full spectra dataset is reported in Figure S4a. Ce^4+^ (red line/squares) and Ce^3+^ (blue line/squares) (**b**) spectral component and (**c**) concentration profiles extracted from unbiased MCR-ALS routine (99.6% of variance explained). CeO_2_ and CeF_3_ reference spectra are reported in the top inset with red and blues line, respectively.

To quantify cerium reduction, Ce M_5_-edge AP-NEXAFS spectra were collected under the same conditions employed for the IR experiment, i.e., H_2_-TPR was performed after having heated the sample for 30 minutes at 300 °C under O_2_:He. Starting with C100, the as-prepared material presented a spectrum (Figure 3a) comparable to reference CeO_2_ (Figure 3b inset). However, during heating under He:H_2_ (Figure S8a), the Ce^4+^ bands initially gained intensity and lost the shoulder at 891 eV. The presence of this shoulder suggested a minor contribution of Ce^3+^ in C100 after oxidation. At T > 200 °C, the main edge lost intensity again, while a two-band shoulder arose at around 891 eV. These bands became structured around 300 °C, and at 350 °C they had a final shape clearly attributable to Ce^3+^. As we recently reported, Ce^3+^/Ce^4+^ can be quantified from M_5_-edge NEXAFS spectra with a driven MCR-ALS protocol where 10 replicas of CeO_2_ and CeF_3_ references spectra are added at the end of the dataset [40]. This method allowed to improve the identification of principal components whilst simultaneously adapting the references to the dataset. The procedure identified two principal components (Figure 3b) describing 99.6% of the variance. The component spectra were clearly related to the pure spectra of Ce^4+^ and Ce^3+^, though with a band width specifically related to these samples. Moreover, the CeO_2_ concentration profiles reported in Figure 3c indicated an evolution very close to the one observed in FT-IR experiments. We noticed that a minor content of Ce^3+^ (≈8%) was already present in the sample after oxidation which completely disappeared during heating. Ce^3+^ was then formed again at T > 200 °C and it reached levels of 10% and 30% at 300 °C and 350 °C, respectively. Even though the initial 8% of Ce^3+^ is within the MCR-ALS protocol error, we clearly observed that Ce^3+^ fingerprints were already present in C100 at RT (Figure S8b), confirming the reliability of the performed quantification.

Interestingly, since the FT-IR absorbance of the Ce^3+^ band and Ce M_5_-edge NEXAFS results followed the same trend, we attempted to extract the Ce^3+ 2^F_5/2_→^2^F_7/2_ transition molar attenuation coefficient. By exploiting the integrated Beer–Lambert law [61] (Equation (2) where A = absorbance, ε = molar attenuation coefficient, c = Ce^3+^ concentration, w = Ce content, and S = pellet area), we reported for the same temperature the evaluated Ce^3+^ concentration (through Ce M_5_-edge fit) with respect to the Ce^3+^ FT-IR band integrated area (Figure S9).

(2) $$A\left(cm^{-1}\right)=\varepsilon \left(\frac{cm}{\mu mol\_Ce}\right)*c\left(wt\%\;Ce^{3+}\right)*\frac{w\left(\mu mol_{Ce}\right)}{S\left(cm^{2}\right)}$$

The slope of the scatter plot linear fit (Figure S9) indicated that ε=0.39±0.02 cm/μmol_Ce. This approach is conventionally used for determining ε of adsorbed species [62,63,64,65]. In contrast, this is so far the first attempt to evaluate the Ce^3+^ molar extinction coefficient and it could potentially be used in the future to evaluate Ce^3+^ concentration from FT-IR measurements. 

**Figure 4:**
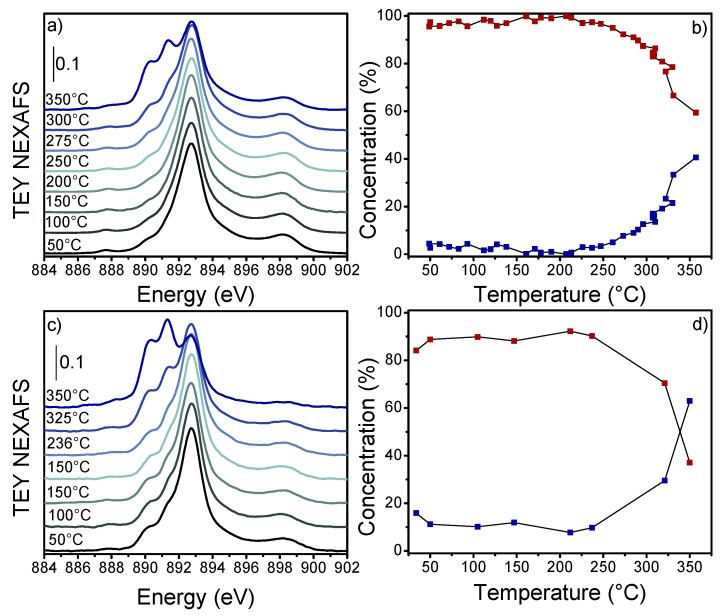
(**a**) C50Z50 and (**c**) C5Z95 Ce M_5_-edge NEXAFS spectra measured during heating under 50 mL/min H_2_:He (3:2) from RT (black line) to 350 °C (blue line). The full spectra dataset is reported in Figure S10. (**b**) C50Z50 and (**d**) C5Z95 concentration profiles of Ce^4+^ (red squares) and Ce^3+^ (blue squares) obtained from MCR-ALS protocol applied to the experimental spectra in panels (**a**,**c**). CeO_2_ and CeF_3_ reference spectra are reported (Figure 3b).

Regarding the mixed oxides, cerium showed a higher reducibility to Ce^3+^ with an increase in Zr content. In particular, we noticed that C50Z50 and C5Z95 presented 5 and 18% of Ce^3+^ in the prepared sample, respectively. At 350 °C under H_2_, the Ce^3+^ content increased to 40% for C50Z50 and 60% for C5Z95 (Figure 4). Indeed, it is well known that Ce reducibility increases in CeZrO_x_ solid solutions due to lattice straining induced by the different ionic radius of Zr [1,66]. 

It is noteworthy that the latter sample contained ≈18% of Ce^3+^ at 150 °C, confirming that the significant t-OH hypsochromic shift (Figure S7f) observed at this temperature was related to the high Ce^3+^ content (Figure 4c). Moreover, by combining the calculated ε with C50Z50 integrated absorbance after H_2_-TPR at 300 °C (≈1.09 cm^−1^, Figure S7c), we calculated a Ce^3+^ ≈ 14%, in agreement with the 13.6% of Ce^3+^ evaluated from the Ce M_5_-edge NEXAFS at the same temperature (Figure 4b).

## 4. Conclusions

Ce/Zr-UiO-66 calcination was presented as a cheap and simple synthesis pathway to obtain nanoparticles of CeO_2_ and homogeneous CeZrO_x_ solid solutions. The MOF calcination temperature was determined by TG analysis whilst PXRD and EDX measurements confirmed a Ce/Zr homogenous dispersion. Due to their nanosize and homogeneity, the obtained oxides are ideal candidates for a deep understanding of their FTIR and NEXAFS spectra properties under reducing conditions. Cerium reduction occurred at RT under H_2_ and it was related to the use of a MOF as a precursor. Moreover, Ce reducibility increased with the Zr content. A careful analysis of CeO_2_ FT-IR H_2_-TPR spectra unveiled that the Ce^3+ 2^F_5/2_→^2^F_7/2_ transition can be used to monitor CeO_2_ bulk reduction. Moreover, we reported that the ν(OH) hypsochromic shift can be used to qualitatively determine the absence/presence of Ce^3+^-V_O_ sites on the catalyst surface. Ce^3+^ was quantified by applying the MCR-ALS protocol to in situ Ce M_5_-edge NEXAFS spectra. NEXAFS results reproduced the infrared results, hence confirming the reliability of the latter. 

Eventually, by combining CeO_2_ FTIR and Ce M_5_-edge NEXAFS spectra, the Ce^3+ 2^F_5/2_→^2^F_7/2_ molar absorption coefficient was calculated. The coefficient was further used to calculate Ce^3+^ content in mixed CeZrO_x_, leading to results in line with Ce M_5_-edge NEXAFS quantification. This proved that the determined molar absorption coefficient value could be further employed for Ce^3+^ quantification during operational FT-IR experiments.

We then demonstrated that the CeO_2_ FTIR spectrum presents excellent markers to extract valuable information on the reduction state of bulk and surface Ce^3+^. These fingerprints can be potentially monitored under relevant reaction conditions with a time resolution an order of magnitude faster than NEXAFS (seconds vs. minutes). 

Nevertheless, the integrated area of the Ce^3+^ band and ν(OH) vibration are easily disturbed in case of doped Ce-based solid solutions (i.e., CeZrO_x_) where, depending on Ce content and its dispersion, only one of the two was meaningful. On the contrary, Ce M_5_-edge NEXAFS spectra were sensitive to Ce even with loading ≈ 5%, giving the technique access to all the possible combinations of Ce-based materials. 

## Data Availability

Not applicable.

## Supplementary Materials

The following supporting information can be downloaded at: https://www.mdpi.com/article/10.3390/nano13020272/s1. Table S1. Employed reactants quantities for the MOF synthesis. Table S2. Quantities of MOFs employed and quantities of oxides obtained after MOFs calcination. Table S3. Elemental composition, textural and structural properties of the six samples. ^a^ ICP results. ^b^ EDX results. Figure S1. Thermal protocol employed for (a) O_2_-TPO and (b) H_2_-TPR measurements. Figure S2. PXRD pattern of (a) C100-UiO-66 (red line), C50Z50-UiO-66 (green line) and C5Z95-UiO-66 (blue line) and (c) CeO2 (red line), C50Z50 (green line) and C5Z95 (blue line). Detail of fcu and Fm-3m main Bragg reflections are reported in panels (b,d), respectively. Figure S3. (a) C100, (b) C50Z50 and (c) C5Z95 PXRD experimental data (black line), refined pattern (red line) and difference function (blue line). Figure S4. N_2_ adsorption/desorption isotherms of (a) C100-UiO-66 (red line), C50Z50-UiO-66 (green line) and C5Z95-UiO-66 (blue line) and (b) C100 (red line), C50Z50 (green line) and C5Z95 (blue line). Figure S5. FT-IR spectra collected on C100 during the: (a) protocol step I (temperature rises from black to green line), (b) O_2_-TPR (temperature rises from black to red line) and (c) H2-TPR (temperature rises from balck to blue line). Detail of ν(OH) and Ce^3+ 2^F_5/2_→^2^F_7/2_ electronic transtion are reported in the smaller panels indictaed with ’) and ’’), respectively. Figure S6. Examples of possible OH groups species potentially formed over CeO_2_ and CeZrO_x_. surface. Ce^4+^ and Ce^3+^ atoms are represented with red and blue colours, respectively. Figure S7. FTIR spectra collected during H_2_-TPR experiment on (a–c) C50Z50 and (d–f) C5Z95 samples. Detail of (a,d) ν(OH) and (b,e) Ce^3+ 2^F_5/2_→^2^F_7/2_ regions (temperature increases from black to blue line). (c) C50Z50 Ce^3+ 2^F_5/2_→^2^F_7/2_ integrated area (black squares) respect to temperature evolution (red squares). (f) C5Z95 t-OH position (blue line) respect to temperature evolution (red squares). Figure S8. C100 (a,b) Ce M5 edge NEXAFS spectra collected during H_2_-TPR experiment. Temperatures are reported in the graph legend. Figure S9. (a) Ce3+ concentration evaluated by Ce M_5_-edge NEXAFS fit reported with respect to the Ce^3+^ FT-IR band integrated area collected at the same temperature. Linear fit is reported with red line whilst its equation and the Pearson R value are reported in the graph. (b) Residual plot for the employed linear fit model. Figure S10. (a) C50Z50 and (b) C5Z95 Ce M_5_-edge NEXAFS spectra measured during heating under 50 mL/min H_2_:He (3:2). Temperature increases from black to blue line. References [51,52,53,54,55,67] are cited in supplementary materials.
