# Supplementary material for: MOF-Derived CeO2 and CeZrOx Solid Solutions: Exploring Ce Reduction through FTIR and NEXAFS Spectroscopy
Source: Nanomaterials (Basel). 2023 Jan 9;13(2):272. doi: 10.3390/nano13020272 (PMC9865843; doi:10.3390/nano13020272)
Supplement: Supplementary file 1 [file nanomaterials-13-00272-s001.zip › nanomaterials-2135456-supplementary.pdf]

MOF-Derived CeO_2 and $CeZrO_x$ Solid Solutions: Exploring Ce Reduction through FTIR and NEXAFS Spectroscopy

Davide Salusso [1,2], Silvia Mauri [3,4], Gabriele Deplano [1], Piero Torelli [3], Silvia Bordiga [1] and Sergio Rojas-Buzo [1,*]

[1] Department of Chemistry, NIS Center and INSTM Reference Center, University of Turin, 10125 Turin, Italy; davide.salusso@unito.it (D.S.); gabriele.deplano@unito.it (G.D.); silvia.bordiga@unito.it (S.B.)
[2] European Synchrotron Radiation Facility, CS 40220, CEDEX 9, 38043 Grenoble, France
[3] IOM CNR Laboratorio TASC, AREA Science Park, Basovizza, 34149 Trieste, Italy; mauri@iom.cnr.it (S.M.); piero.torelli@elettra.eu (P.T.)
[4] Department of Physics, University of Trieste, Via Valerio 2, 34127 Trieste, Italy
* Correspondence: sergio.rojasbuzo@unito.it

Supporting information

Table of contents

1. Materials and Methods 2
2. Structural and textural properties 3
3. Sample activation procedure 5
4. FT-IR and NEXAFS H_2-TPR 6
a. Details on molar absorption coefficient calculation 7

1. Materials and Methods

Table S1. Employed reactants quantities for the MOF synthesis.

Sample	DMF (mL)	Ce solution (mL)	Zr solution (mL)
C100-UiO-66	9.00	3.00	-
C50Z50-UiO-66	7.20	1.78	0.59
C5Z95-UiO-66	7.20	0.20	2.16

Table S2. Quantities of MOFs employed and quantities of oxides obtained after MOFs calcination.

Sample	MOF weight (mg)	Derived-oxide weight (mg)
C100-UiO-66	357	147
C50Z50-UiO-66	235	92
C5Z95-UiO-66	363	126

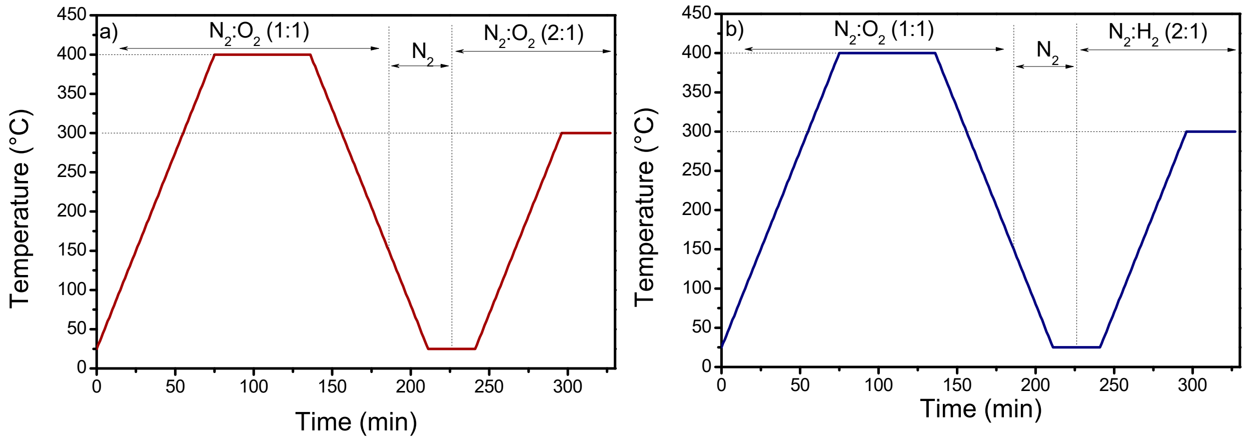

Figure S1. Thermal protocol employed for a) O_2-TPO and b) H_2-TPR measurements.

2. Structural and textural properties

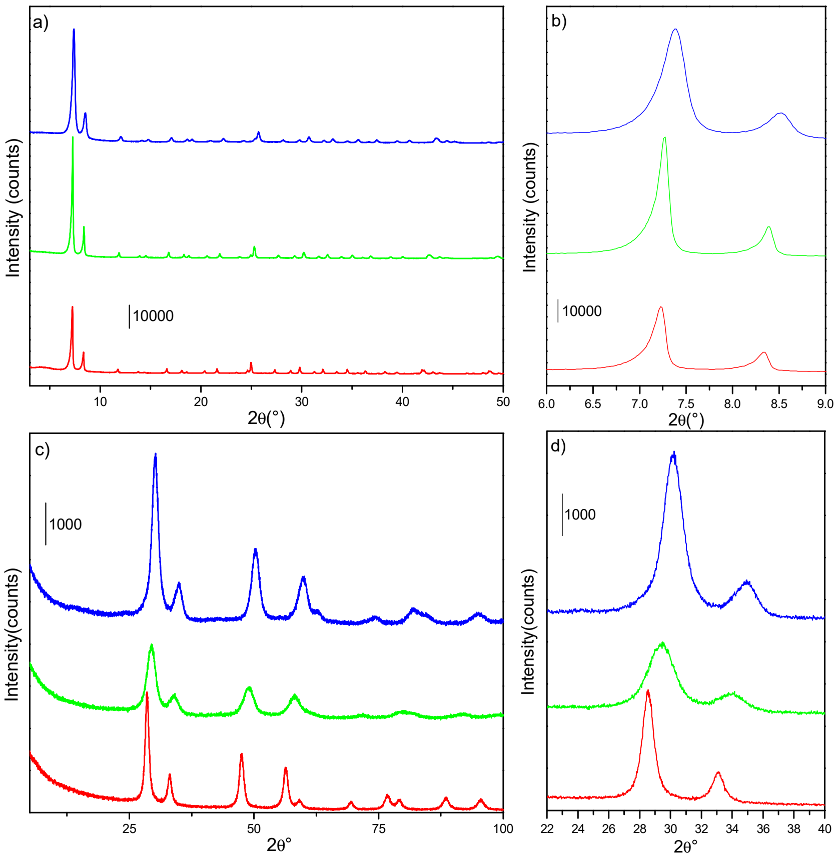

Figure S2. PXRD pattern of a) C100-UiO-66 (red line), C50Z50-UiO-66 (green line) and C5Z95-UiO-66 (blue line) and c) CeO_2 (red line), C50Z50 (green line) and C5Z95 (blue line). Detail of *fcu* and Fm-3m main Bragg reflections are reported in panels b and d, respectively.

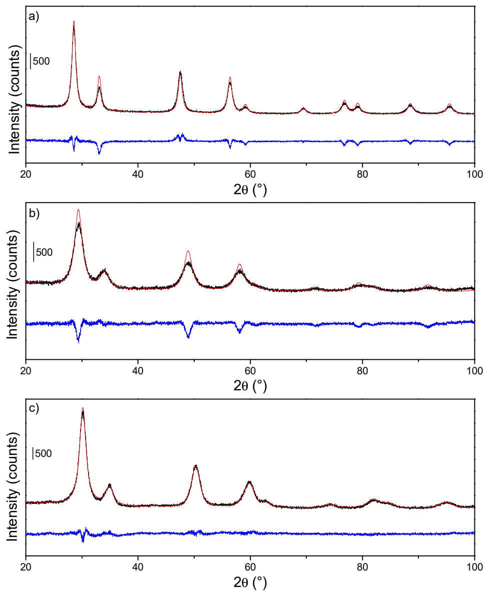

Figure S3. a) C100, b) C50Z50 and c) C5Z95 PXRD experimental data (black line), refined pattern (red line) and difference function (blue line).

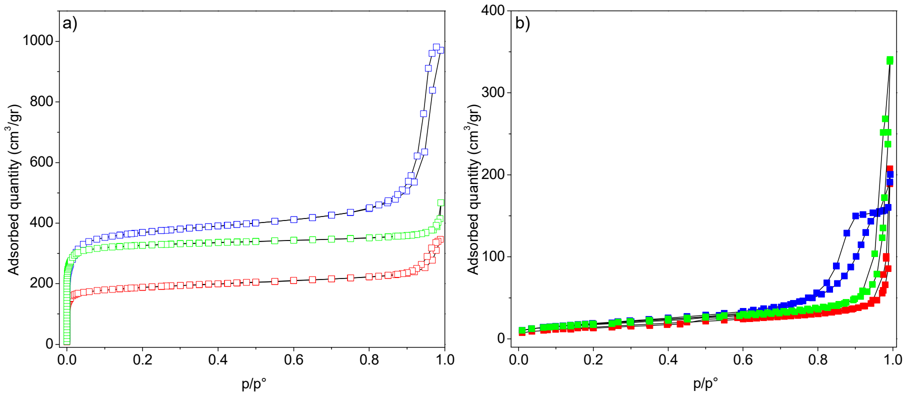

Figure S4. N_2 adsorption/desorption isotherms of a) C100-UiO-66 (red line), C50Z50-UiO-66 (green line) and C5Z95-UiO-66 (blue line) and b) C100 (red line), C50Z50 (green line) and C5Z95 (blue line).

Table S3. Elemental composition, textural and structural properties of the six samples. [a] ICP results. [b] EDX results.

	C100-UiO-66	C50Z50-UiO-66	C5Z95-UiO-66	C100	C50Z50	C5Z95
Ce:Zr	/	43:57[a]	4:96[a]	/	49:51[b]	5:95[b]
SSA (m^2/g)	1080	1387	1277	54	67	70

Approximative Crystallite size (nm)	/	/	/	8	4	5

3. Sample activation procedure.

FT-IR spectra collected over C100 (C50Z50 and C5Z95 are not reported for brevity) during the measurement protocol step I (Figure S5a) showed that increasing the temperature under oxidising atmosphere causes a loss of adsorbed water highlighted by the decrease of H-bonded OH group contribution (≈3400 cm^{-1}) together to a consumption of carbonates (1300-1600 cm^{-1}) and organic compounds (2800-3000 cm^{-1}). Parallel to water loss the hydroxyl groups become isolated and vibrate to more specific frequencies, forming three bands at 3705, 3684 and 3659 cm^{-1}, associated to terminal (m-OH), bridged (b-OH) and tri-bridged (t-OH) hydroxyl groups, respectively, sketched in Figure S6. A further temperature decrease to 25°C caused adsorption of some H_2O molecules on the catalyst surface, as observable from the partial regeneration of the H-bonded broad band centred at 3300 cm^{-1} however, without affecting the isolated OH groups.

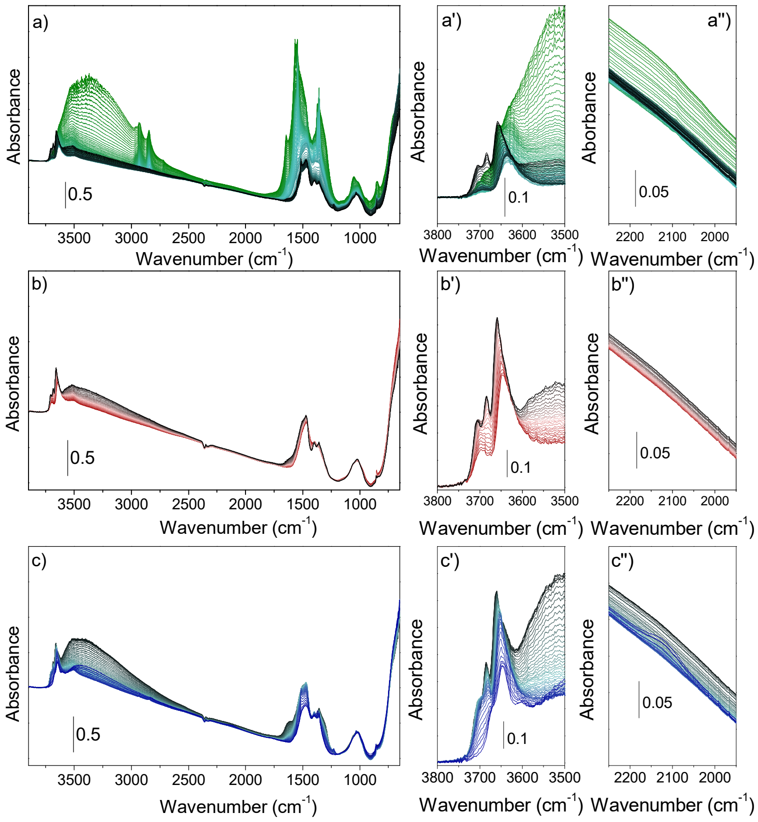

Figure S5. FT-IR spectra collected on C100 during the : a) protocol step I (temperature rises from black to green line), b) O_2-TPR (temperature rises from black to red line) and c) H_2-TPR (temperature

rises from balck to blue line). Detail of ν(OH) and Ce^{3+} $^2F_{5/2}$→$^2F_{7/2}$ electronic transtion are reported in the smaller panels indictaed with ') and ''), respectively.

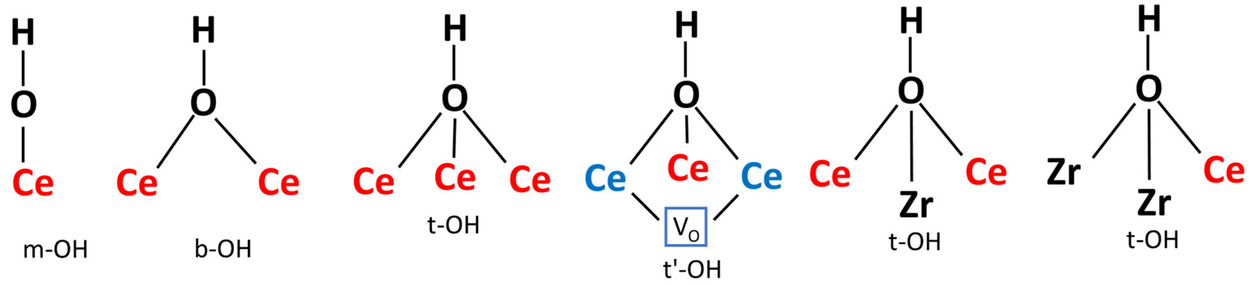

Figure S6. Examples of possible OH groups species potentially formed over CeO_2 and $CeZrO_x$. surface. Ce^{4+} and Ce^{3+} atoms are represented with red and blue colours, respectively.

4. FT-IR and NEXAFS H_2-TPR

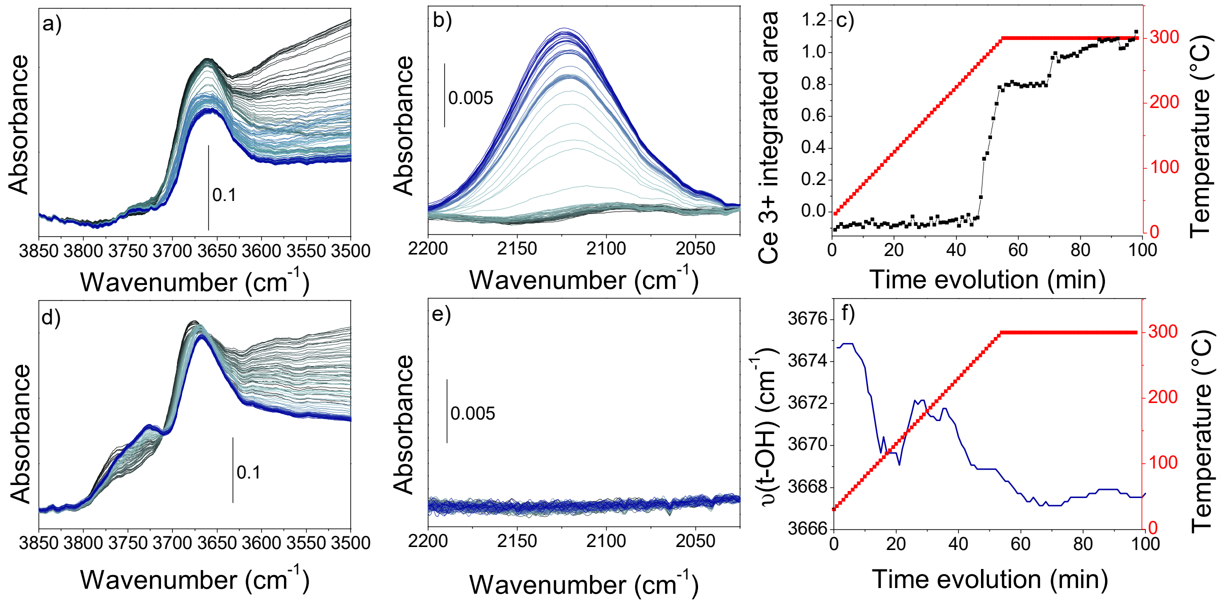

Figure S7. FTIR spectra collected during H_2-TPR experiment on a,b,c) C50Z50 and d,e,f) C5Z95 samples. Detail of a,d) ν(OH) and b,e) Ce^{3+} $^2F_{5/2}$→$^2F_{7/2}$ regions (temperature increases from black to blue line). c) C50Z50 Ce^{3+} $^2F_{5/2}$→$^2F_{7/2}$ integrated area (black squares) respect to temperature evolution (red squares). f) C5Z95 t-OH position (blue line) respect to temperature evolution (red squares).

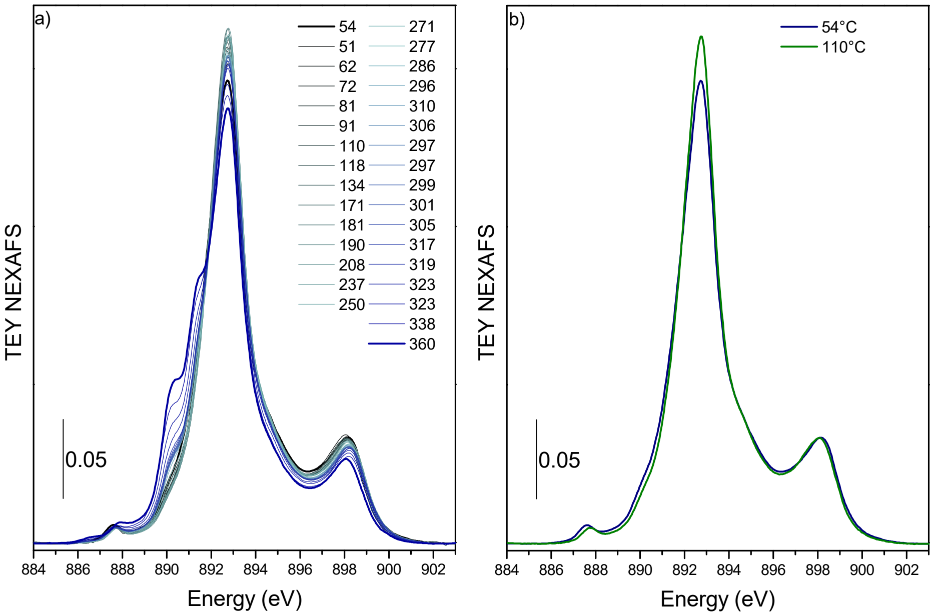

Figure S8. C100 a,b) Ce M_5 edge NEXAFS spectra collected during H_2-TPR experiment. Temperatures are reported in the graph legend.

a. Details on molar absorption coefficient calculation

For determining the molar absorption coefficient of Ce^{3+} $^2F_{5/2}\rightarrow{}^2F_{7/2}$ transition we have employed the integrated version of the Beer-Lambert Law (BLL):

$$A(cm^{-1}) = \varepsilon\left(\frac{cm}{\mu mol}\right) * c(wt\%\ Ce^{3+}) * \frac{w(\mu mol)}{S(cm^2)}$$

It should be point out as in IR spectroscopy the BLL is usually employed for determining either the concentration or the molar absorption coefficient (ε) of an adsorbate over a support. On the contrary we here attempted to determine the ε of a band arising from Ce electronic transition. For this reason, the BLL was expressed in function of Ce content in the employed pellet. Moreover, since the transition is specific for Ce^{3+}, the concentration was expressed as Ce^{3+} wt%. Ce^{3+} concentration (weighted for Ce content "W" and pellet area "S") and Ce^{3+} $^2F_{5/2}\rightarrow{}^2F_{7/2}$ band integrated area measured at the same temperature were reported in a scatter plot (Figure S9a). The ε value was determined from the slope of the linear fit (red line Figure S9a). The residuals analysis (Figure S9b) showed random distribution of their values, which combined with the Pearson correlation of 0.97, confirmed the robustness of the model.

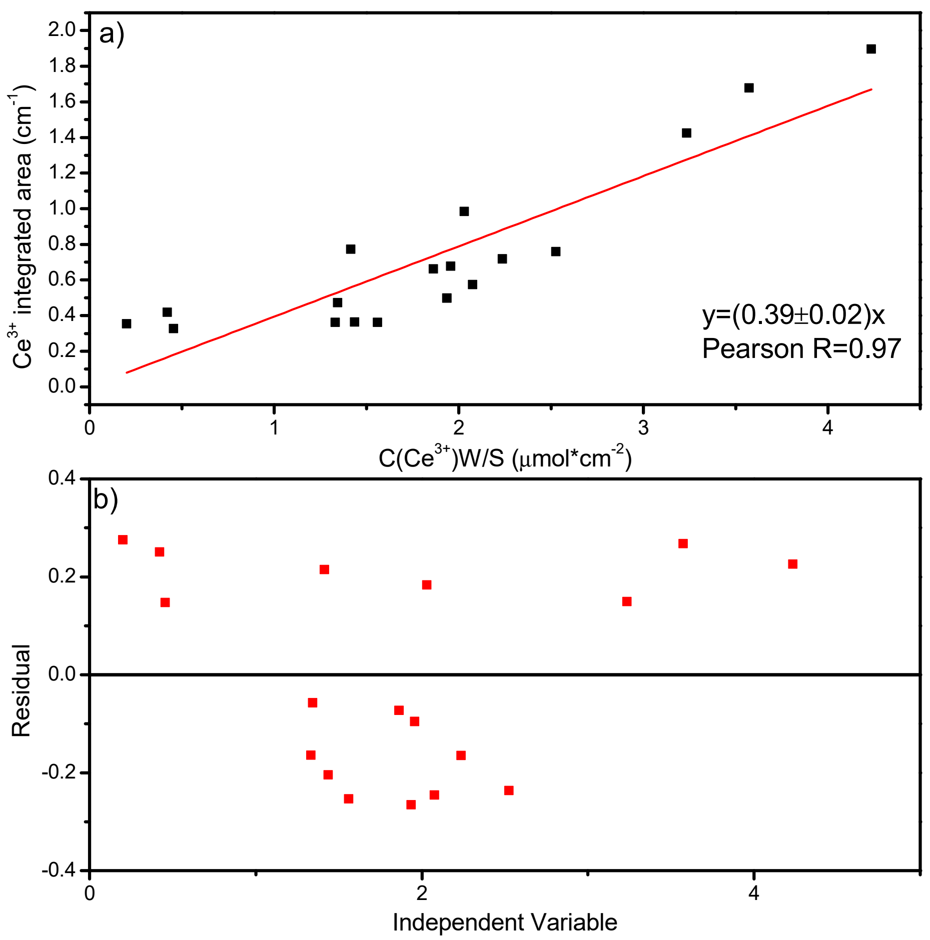

Figure S9. a) Ce^{3+} concentration evaluated by Ce M_5-edge NEXAFS fit reported with respect to the Ce^{3+} FT-IR band integrated area collected at the same temperature. Linear fit is reported with red line whilst its equation and the Pearson R value are reported in the graph. b) Residual plot for the employed linear fit model.

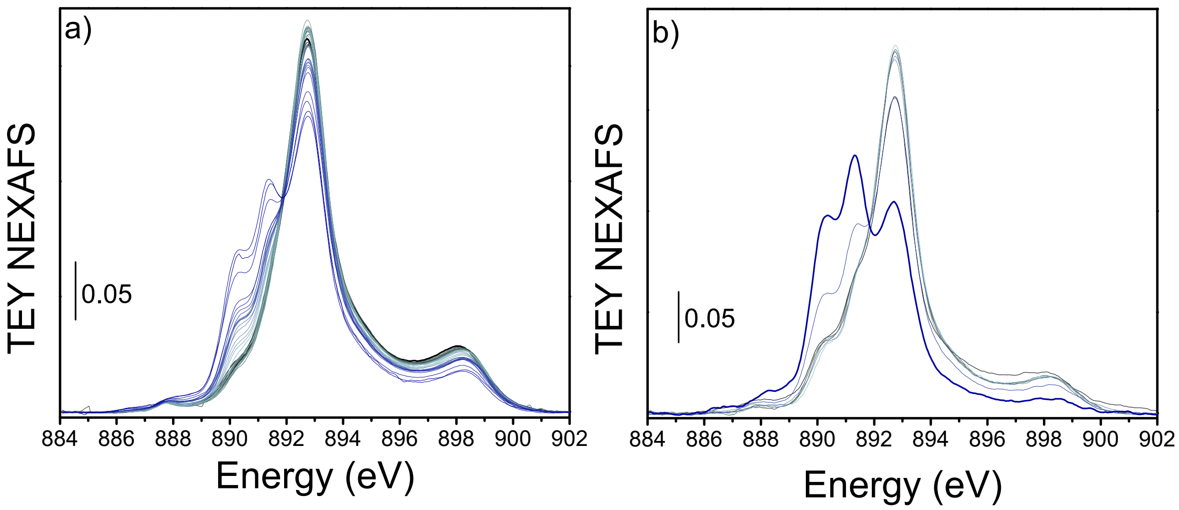

Figure S10. a) C50Z50 and b) C5Z95 Ce M_5-edge NEXAFS spectra measured during heating under 50 mL/min H_2:He (3:2). Temperature increases from black to blue line.

References

1. Lammert, M.; Glißmann, C.; Stock, N. Tuning the Stability of Bimetallic Ce(Iv)/Zr(Iv)-Based MOFs with UiO-66 and MOF-808 Structures. *Dalt. Trans.* **2017**, *46*, 2425–2429. https://doi.org/10.1039/c7dt00259a.
2. Rodríguez-Carvajal, J. Recent Developments of the Program Fullprof. *Newsl. Comm. Powder Diffr. IUCr* **2001**, *26*, 12–19.
3. Thompson, P.; Cox, D.E.; Hastings, J.B. Rietveld Refinement of Debye-Scherrer Synchrotron X-Ray Data from Al_2O_3. *J. Appl. Crystallogr.* **1987**, *20*, 79–83.
4. Simonne, D.; Martini, A.; Signorile, M.; Piovano, A.; Braglia, L.; Torelli, P.; Borfecchia, E.; Ricchiardi, G. THORONDOR: Software for Fast Treatment and Analysis of Low-Energy XAS Data. *J. Synchrotron Radiat.* **2020**, *27*, 1741–1752. https://doi.org/10.1107/S1600577520011388.
5. Ruckebusch, C.; Blanchet, L. Multivariate Curve Resolution: A Review of Advanced and Tailored Applications and Challenges. *Anal. Chim. Acta* **2013**, *765*, 28–36. https://doi.org/10.1016/j.aca.2012.12.028.
6. Ravel, B.; Newville, M. ATHENA, ARTEMIS, HEPHAESTUS: Data Analysis for X-Ray Absorption Spectroscopy Using IFEFFIT. *J. Synchrotron Radiat.* **2005**, *12*, 537–541. https://doi.org/10.1107/S0909049505012719.
